# The olfactory bulb coordinates the ventral hippocampus–medial prefrontal cortex circuit during spatial working memory performance

**DOI:** 10.1186/s12576-022-00833-5

**Published:** 2022-04-25

**Authors:** Morteza Salimi, Farhad Tabasi, Milad Nazari, Sepideh Ghazvineh, Mohammad Reza Raoufy

**Affiliations:** 1 Department of Physiology, Faculty of Medical Sciences, Tarbiat Modares University, Tehran, Iran; 2 Institute for Brain Sciences and Cognition, Faculty of Medical Sciences, Tarbiat Modares University, Tehran, Iran; 3 Department of Molecular Biology and Genetics, Aarhus University, Aarhus, Denmark; 4 DANDRITE, The Danish Research Institute of Translational Neuroscience, Aarhus University, Aarhus, Denmark; 5 Center for Proteins in Memory-PROMEMO, Danish National Research Foundation, Aarhus, Denmark

**Keywords:** Olfactory bulb, Ventral hippocampus, Medial prefrontal cortex, Working memory, Functional connectivity

## Abstract

Neural oscillations synchronize the activity of brain regions during cognitive functions, such as spatial working memory. Olfactory bulb (OB) oscillations are ubiquitous rhythms that can modulate neocortical and limbic regions. However, the functional connectivity between the OB and areas contributing to spatial working memory, such as the ventral hippocampus (vHPC) and medial prefrontal cortex (mPFC), is less understood. Hence, we investigated functional interaction between OB and the vHPC–mPFC circuit during the spatial working memory performance in rats. To this end, we analyzed the simultaneously recorded local field potentials from OB, vHPC, and mPFC when rats explored the Y-maze and compared the brain activities of correct trials vs. wrong trials. We found that coupling between the vHPC and mPFC was augmented during correct trials. The enhanced coherence of OB activity with the vHPC–mPFC circuit at delta (< 4 Hz) and gamma (50–80 Hz) ranges were observed during correct trials. The cross-frequency analysis revealed that the OB delta phase increased the mPFC gamma power within corrected trials, indicating a modulatory role of OB oscillations on mPFC activity during correct trials. Moreover, the correlation between OB oscillations and the vHPC–mPFC circuit was increased at the delta range during correct trials, exhibiting enhanced synchronized activity of these regions during the cognitive task. We demonstrated a functional engagement of OB connectivity with the vHPC–mPFC circuit during spatial working memory task performance.

**Supplementary Information:**

The online version contains supplementary material available at 10.1186/s12576-022-00833-5.

## Introduction

Working memory is a short-term system for holding and manipulating newly acquired information from the environment or retrieving information from long-term memory [1]. Several mammalian brain regions, particularly the prefrontal cortex and hippocampus, are involved in working memory processes [1, 2]. Hence, coordinated communication between the medial prefrontal cortex (mPFC) and ventral hippocampus (vHPC) is essential for successful spatial working memory performance [3–6]. It has been demonstrated that vHPC–mPFC functional connectivity is enhanced during a cognitive function, such as working memory [3, 7, 8]. Moreover, this circuit has a close functional interaction with other brain regions to organize their processes, in which their communications are facilitated by synchronized oscillations [9–11].

Brain oscillations are known as a reflection of activities pertaining to various brain functions, including cognitive performance, such as working memory [12–15]. For instance, working memory is correlated with enhanced theta range oscillations activity in mPFC and vHPC [16–19]. Moreover, we have previously shown that coupling between theta and gamma oscillations in the olfactory bulb (OB) is critical for correct working memory performance [20].

The olfactory bulb (OB) is a key brain region, which is critically implicated in a range of cognitive functions [21–23], especially memory [24–26]. The OB is anatomically connected with other brain structures associated with memory processes; it is linked to the hippocampal formation through the entorhinal cortex [27] and reciprocally receives direct synapses from the ventral region of HPC [28]. Furthermore, OB and mPFC have both structural and functional connections during cognitive performances [23, 29, 30].

The OB activity is one important source of ubiquitous brain oscillations propagating in the brain, and importantly, are engaged in network synchronization [31]. These brain rhythms can be generated independently from an odor stimulation: it has been demonstrated that olfactory sensory neurons (OSNs) have a dual function [32]. They not only can respond to odor stimulus but can be triggered by the passage of odor-free air during nasal respiration, subserving as mechano-receptors [32]. Activated OSNs by airflow increase these neurons' firing rate, generate rhythmic electrical activities in the olfactory system, and most likely drive theta oscillations in phase with respiration [32]. These activities are diminished when the current of air is diverted from nose to mouth, or by intubation, in both humans and rats [30, 33, 34]. These respiratory-entrained oscillations are suggested as a synchronizing activity for brain networks during cognitive processes [25, 31, 35].

For instance, hippocampal respiratory rhythm (HRR) is an olfactory system-entrained oscillation in the hippocampus [36–39] that is implicated in cognitive performance [37, 39]. Moreover, OB oscillations can modulate the neuronal firing rate and activity of mPFC at delta and theta frequencies [40–42]. Like HRR, the olfactory system drives another rhythmic activity in the prefrontal area known as prefrontal respiratory rhythm (PRR), generated by OSNs during nasal respiration [42]. It has been suggested that PRR contributes to information processing in the prefrontal neuronal network and is essential for cognition [29, 42]. Moreover, removing or inhibiting the OB results in impairment of several critical cognitive functions, such as attentional tasks, reference memory, delayed matching, reversal memory, and working memory deficits [43–45].

Although previous studies have investigated the significance of OB in cognitive performance and its interaction with the activity of distant brain areas [30, 32, 46], the communication of OB with the vHPC–mPFC circuit during working memory performance is not elucidated yet. Given rich anatomical connections between OB, mPFC, and vHPC structures, and considering previous evidence regarding the role of OB activities on synchronized brain activities in cognitive performance, we hypothesized that OB could modulate vHPC–mPFC circuit activity during spatial working memory task performance. Therefore, to address whether OB oscillations can modulate vHPC–mPFC rhythmical activities in association with the cognitive process, we explored the functional connectivity of the OB–vHPC–mPFC network when rats performed the spatial working memory task in a Y-maze.

## Materials and methods

### Animals

Six adults (2–3 months) pathogen-free male Wistar rats weighing 210–230 g were obtained from Tarbiat Modares University (Tehran, Iran) and housed at 21 ± 2 °C, 12-h light–dark cycle. Rats were kept in standard animal research facilities, in which food and water were available. The protocol of the study was approved by the “Ethics Committee of Faculty of Medical Sciences, Tarbiat Modares University.”

### Electrode implantation and histological verification

Animals were anesthetized with intraperitoneal injections of ketamine (100 mg/kg) and xylazine (10 mg/kg). Then, anesthetized rats were placed in a stereotaxic apparatus, and a longitudinal incision was carried out, the skin was drawn back, and the skull was exposed. After drilling the skull, stainless-steel recording electrodes (127 µm in diameter, A.M. system Inc., USA) were implanted unilaterally into stereotaxic coordinates of OB (AP 8.5 mm, ML −1 mm, DV −1.5 mm), mPFC (AP + 3.2 mm; L −0.6 mm; DV −3.6 mm) and vHPC (AP −4.92 mm; L −5.5 mm; DV −7.5 mm) according to the rat brain atlas [47]. Two additional holes were drilled into the skull and used to hold the recording socket on the skull. We also implanted a stainless-steel screw at the right side of the parietal bone as a reference point. The acrylic dental adhesive was poured around the electrodes and bone screw. The skin was sutured, and an antibiotic ointment was used for the wound to prevent infection.

**Fig. 1:**
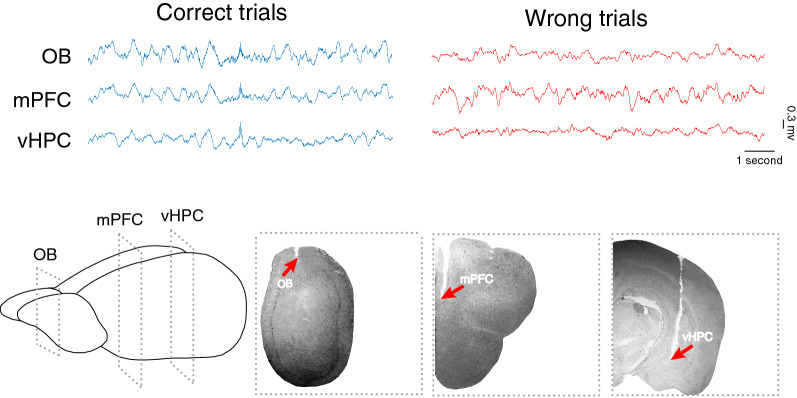
Raw signal and histological verification. (*Top*) Representative raw signals of LFP in OB, mPFC, and vHPC. Blue and red lines denote LFP signals in correct and wrong trials, respectively. (*Bottom*) schematic representation and verified sections of electrode implantation. *LFP* local field potential; *OB* olfactory bulb; *mPFC* medial prefrontal cortex; *vHPC* ventral hippocampus

To verify that electrodes were located at the correct position, we carefully removed rats' brains and fixed them with 4% paraformaldehyde for 48 h. A 200 mm coronal section was visually compared to the matching slices in the rat's brain atlas of Paxinos and Watson (Fig. 1). Three rats with misplaced electrodes and animals that did not perform the Y-maze tasks were excluded from statistical analyses and the entire study. Data presented here are taken from six rats.

### Y-maze test

The Y-maze test was used to assess spatial working memory. Before trials began, all animals were habituated to the behavioral task room for 1 h of 2 consecutive days. Then animals were individually placed into a black Plexiglas Y-maze composed of three identical arms with 120° (length 50, width 10 cm, height 25 cm). Different cues were visible from three arms to facilitate spatial orientation. Rats were placed into one arm's center (Arm A) to freely explore all three arms for 10 min. A ceiling-mounted camera recorded the trials. Since this study was aimed to evaluate the modulatory role of OB oscillations, we only evaluated spontaneous alternation. To avoid any odor cues that may alter the experiment, we used 90% ethanol before and after the task for each animal to decontaminate and remove any odor cues. A correct trial is defined as a movement of the animals to the other two arms without redoing their steps (i.e., Arm A to B to C). A spontaneous alternation such as ABA is considered a wrong trial.

### Signal processing

Local field potentials (LFPs) were simultaneously obtained from OB, vHPC, and mPFC through a fixed socket animal's head connected to a miniature buffer head stage with high-input impedance (BIODAC-A, TRITA Health Technology Co., Tehran, Iran). The signals were amplified (1000 amplification gain), digitized at 1 kHz and low-pass filtered < 250 Hz via AC coupled with the recording system (BIODAC-ESR18622, TRITA Health Technology Co., Tehran, Iran). The signal pre-processing, such as noise rejection and baseline correction, was performed using the EEGlab toolbox [48].

**Fig. 2:**
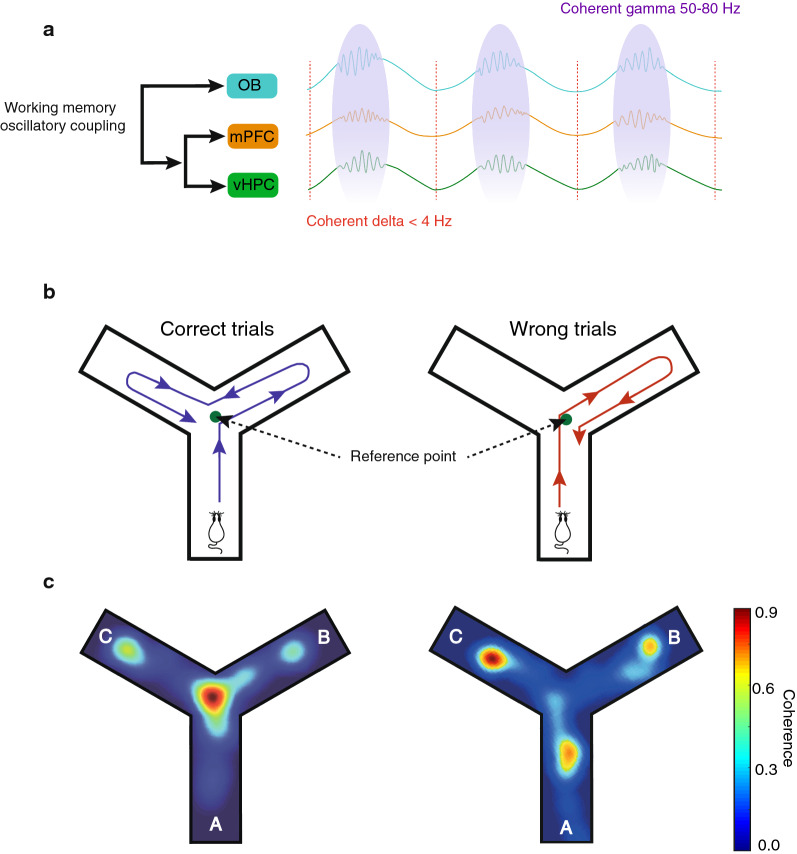
Coherence distribution during working memory performance. **A** Schematic display of OB coordination on the vHPC–mPFC circuit. **B** Schematic illustration showing examples of correct and wrong working memory performance. The correct trials are defined when the animal enters the other two arms without redoing its steps (i.e., Arm A–B–C and ABA is considered a wrong trial). **C** Representative of coherence value when the animal is spontaneously alternating in the Y-maze. LFPs are binned into the positional frame, and the mean pixel coherence is color-coded to generate the coherence on the Y-maze map. Graphs show that the corresponding coherence between OB and mPFC increases when the rat explores the maze center to enter the correct arm (left panel). *LFP* local field potential, *OB* olfactory bulb, *mPFC* medial prefrontal cortex, *vHPC* ventral hippocampus, *RP* reference point

To find whether OB oscillations can modulate vHPC–mPFC rhythms during a cognitive process, we generated inter-regional coherence on a Y-maze map when rats spontaneously alternated between arms. LFP was binned into a positional frame, and the mean pixel coherence was color-coded (Fig. 2). Accordingly, we selected 1 s before animals arrived at the center (defined as the reference point; RP) until they exited the Y-maze center. This time was selected for further analyses. Finally, the coupling parameters during correct trials were averaged per animal and compared with wrong trials.

To calculate the coherence, we computed magnitude-squared coherence using the *mscohere* function of MATLAB. Synchrony of the OB–vHPC–mPFC network signals was measured via cross-correlation analysis, defined as the *xcorr* function in MATLAB software (with the “coeff” option for normalizing values). Cross-frequency coupling (CFC) analysis was performed using phase-power means. Accordingly, the delta phase was calculated by the Hilbert transform, and gamma power was obtained using the spectrogram with one sample time order. Next, the delta phase was binned into the 120 bins with 3°. The average power of gamma samples was then calculated for consecutive bins. Coupling strength was defined as the resultant vector’s length, which was the average of power vectors in the delta phase.

### Statistical analysis

We used GraphPad Prism (version 6.0) for statistical analysis and creating graphs. The normality assessment was performed with the Kolmogorov–Smirnov test within each parameter, and since the distribution was not normal, the Wilcoxson test (as a non-parametric test) was used to compare two groups. The *p* values less than 0.05 were considered statistically significant.

## Results

### OB oscillations are more coherent with vHPC–mPFC circuit activity at delta and gamma range during correct trials.

**Fig. 3:**
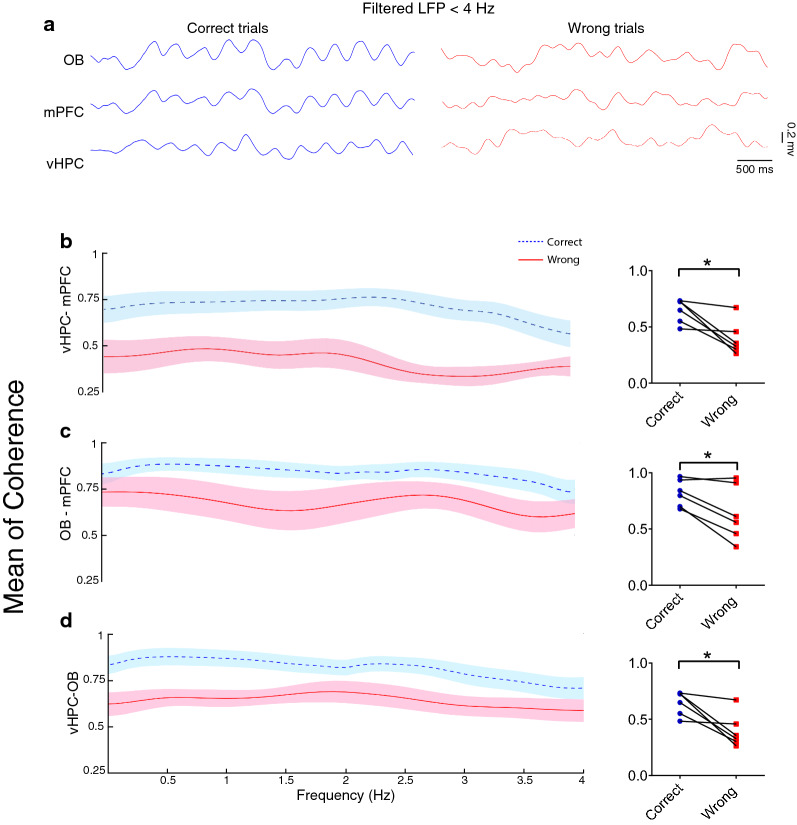
Coherence at the delta band increased during correct working memory performance. **A** Representative sample of filtered LFP signal in the < 4 (Hz) band during correct (blue) and wrong (red). **B**–**D** Coherence of OB, mPFC, and vHPC circuit in delta frequency. Within correct trials, coherence between OB, mPFC, and vHPC was raised compared to wrong trials (data averaged over 24 correct trials, 13 wrong trials 2 s pre and 1 s post the RP). Lines and bar graphs indicate the mean of coherence, and shaded regions and error bars represent SEM. Data were analyzed by Wilcoxson-test, *n* = 6 per group. *p < 0.05, **p < 0.01 and ***p < 0.001. *LFP* local field potential, *OB* olfactory bulb, *mPFC* medial prefrontal cortex, *vHPC* ventral hippocampus, *RP* reference point

The mPFC and vHPC are two critical regions for spatial working memory performance [3] and are structurally connected with direct synaptic pathways [49]. In the delta range (< 4 Hz), we found that vHPC and mPFC were significantly more coherent during correct trials than wrong trials. To investigate the contribution of OB rhythmic activity to working memory, we evaluated OB–mPFC and OB–vHPC coherence. OB coherence with both mPFC and vHPC at delta range was significantly increased on trials in which rats performed the task correctly (Fig. 3).

**Fig. 4:**
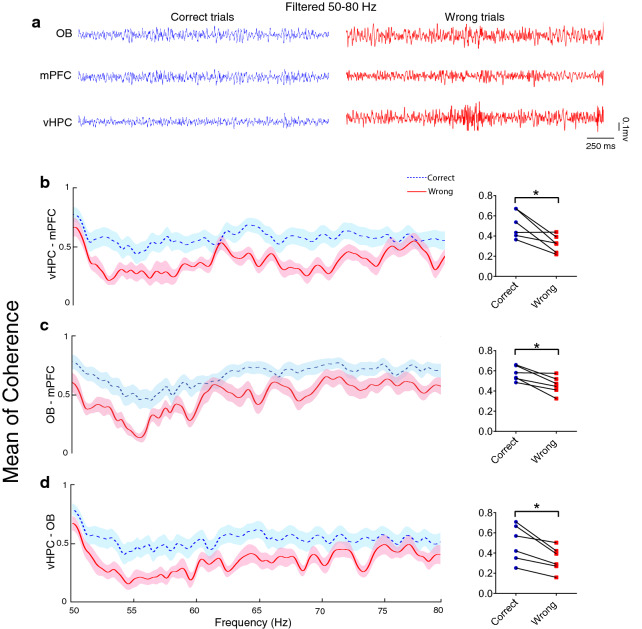
Gamma coherence increased at correct working memory task performance. **A** Representative sample of filtered LFP signal in the 50–80 (Hz) band during correct (blue) and wrong (red). **B–D** Coherence of OB, mPFC, and vHPC circuit in delta frequency. Coherence between OB, mPFC, and vHPC enhanced during correct trials compared to wrong trials (data averaged over 24 correct trials, 13 wrong trials 2 s pre and 1 s post the RP). Lines and bar graphs indicate the mean of coherence, and shaded regions and error bars represent SEM. Data were analyzed by Wilcoxson-test, *n* = 6 per group. *p < 0.05. *LFP* local field potential, *OB* olfactory bulb, *mPFC* medial prefrontal cortex, *vHPC* ventral hippocampus, *RP* reference point

Working memory is associated with enhancement of gamma activity, especially 50–80 Hz in some brain regions of humans and animals [50–52]. Our coherence analysis for gamma oscillations in the OB–vHPC–mPFC network demonstrated a rise at 50–80 Hz of gamma frequency when animals correctly performed working memory performance (see Fig. 4 for more details). However, we did not find noticeable coherence changes of OB–PFC or OB–vHPC at theta (4–12 Hz) and beta (12–30 Hz) frequencies during correct trials (Additional file 1: Figure S1). According to our results of coherence, we applied further analyses in the delta (< 4 Hz) and gamma (50–8 Hz). Altogether, these findings indicate that the OB activity is significantly coherent with the vHPC–mPFC circuit during successful spatial working memory performance.

### *Delta*–*gamma coupling of OB–vHPC–mPFC network enhances during correct spatial working memory task trials.*

**Fig. 5:**
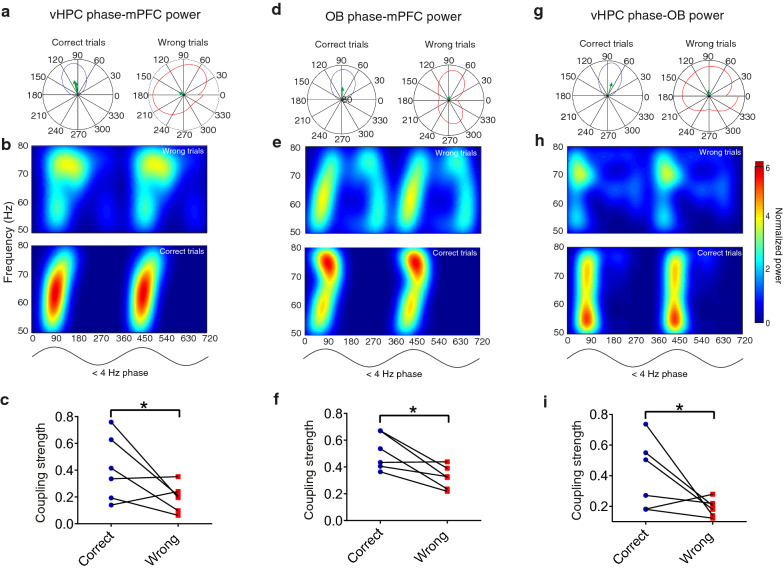
Cross-frequency coupling between OB, mPFC, and vHPC reduces enhanced during the correct trial performance. **A** Polar distribution. **B** Color map of gamma mPFC power (50–80 Hz) and delta phase cycle in vHPC. The green arrow denotes the mean resultant vector length. **C** During correct trials, animals show higher mean resultant vector length values as an indicator of the vHPC delta phase and mPFC gamma power coupling. **D**–**F** Like what is described in **A**–**C**, but for the delta phase of OB and gamma power in mPFC; the delta phase more significantly modulates gamma power in mentioned regions during correct trials than control. **G** Polar distribution. **H** Color map of OB gamma power in along with the vHPC delta phase. **I** Phase-power coupling in the vHPC–OB circuit increased within correct working memory performance. Data were analyzed by Wilcoxson-test, *n* = 6 per group. *p < 0.05, **p < 0.05. *OB* olfactory bulb, *mPFC* medial prefrontal cortex, *vHPC* ventral hippocampus

Previously it has been suggested that low-frequency oscillations, such as delta, modulate high-frequency oscillations, including gamma, during cognitive performance [41, 53]. CFC approach reflects network communication during cognitive processes, such as working memory [3]. Hence, we conducted the phase-power analysis to address whether the phase of delta frequency in one region can modulate another region's gamma power. The resultant vector's length as a phase-power coupling indicator revealed that the coupling between the delta phase of vHPC with the gamma power of mPFC during correct trials is significantly higher than wrong trials (Fig. 5A–C). We also identified that the OB delta phase was coupled with mPFC gamma power during correct trials (Fig. 5D–F). Similarly, OB’s gamma power was significantly modulated by the delta phase of vHPC (Fig. 5G–I). However, we did not observe a significant difference between correct and wrong trials regarding the modulatory effect of the OB delta phase on gamma vHPC power (Additional file 2: Figure S2).

### The OB–vHPC–mPFC synchrony at delta increases during the correct spatial working memory task trials

**Fig. 6:**
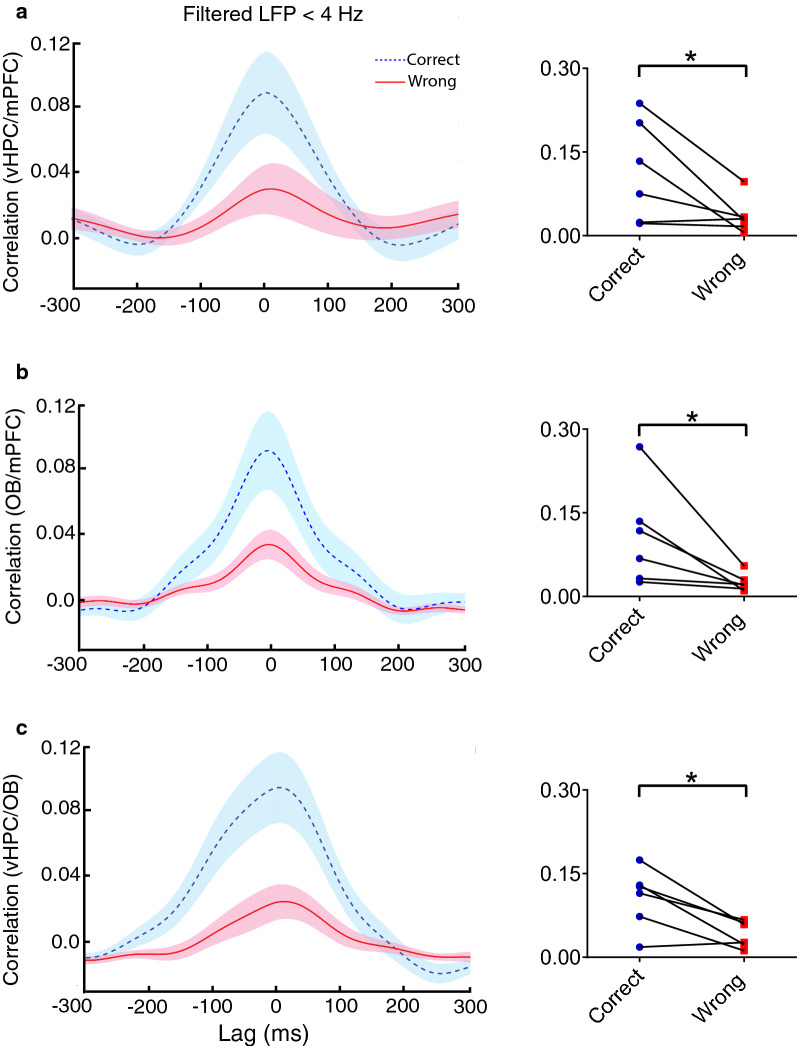
Delta synchrony increased in OB–vHPC–mPFC network when animals correctly performed working memory task. Mean correlation in time lag between **A** vHPC–mPFC, **B** OB–mPFC and **C** vHPC–OB at delta (< 4 Hz) frequency. Correlation coefficient were enhanced during correct trials for delta oscillation. Data were analyzed by Wilcoxson-test, *n* = 6 per group. **p < 0.01, ***p < 0.001 compared to control. *OB* olfactory bulb, *mPFC* medial prefrontal cortex, *vHPC* ventral hippocampus

**Fig. 7:**
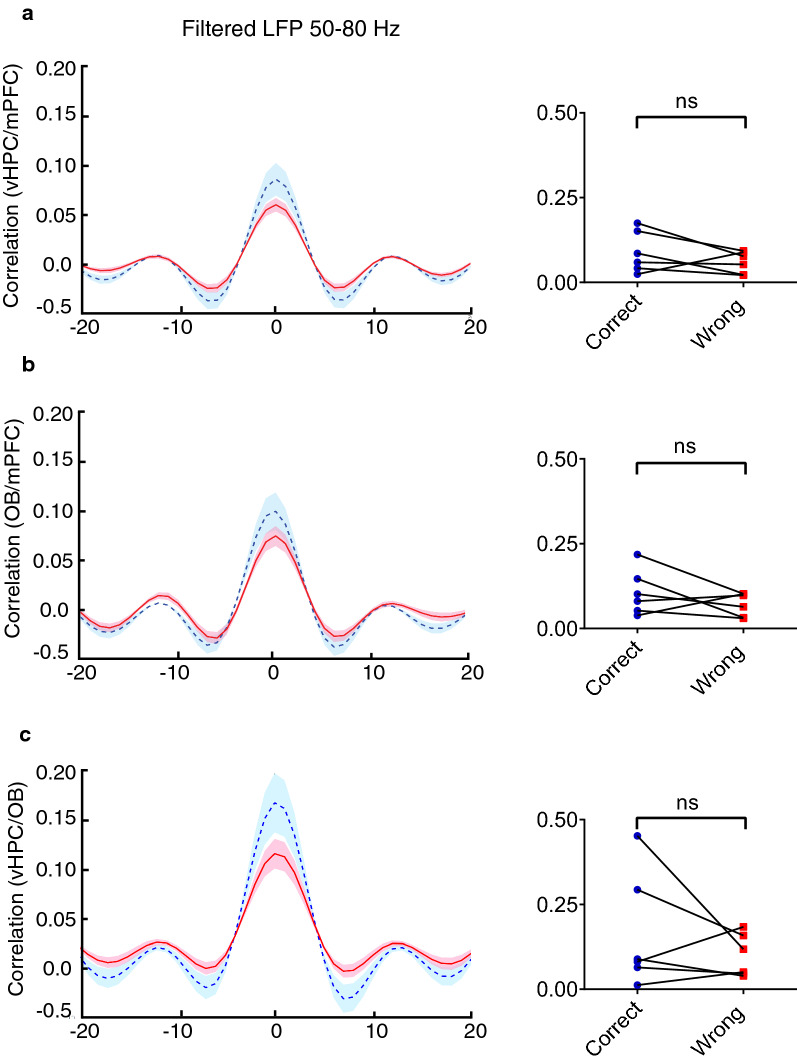
Synchrony in the OB–vHPC–mPFC network at gamma band was not changed. Mean correlation in time lag between **A** vHPC–mPFC, **B** OB–mPFC and **C** vHPC–OB at gamma (50–80 Hz) frequency. Correlation coefficients were not changed during correct vs. wrong Data were analyzed by Wilcoxson-test, *n* = 6 per group. *OB* olfactory bulb, *mPFC* medial prefrontal cortex, *vHPC* ventral hippocampus

We explored the synchrony of the OB–vHPC–mPFC network using cross-correlation analysis. During correct trials, the correlation coefficient between vHPC–mPFC, OB–mPFC, and vHPC–OB at delta frequency (< 4 Hz) was significantly higher compared to wrong trials (Fig. 6). However, in the gamma frequency band (50–80 Hz), no significant synchrony changes were seen in the OB–vHPC–PFC network (Fig. 7). Synchrony in OB–vHPC–mPFC network at delta band may reflect successful response during working memory performance.

## Discussion

Our findings provide an understanding of functional connectivity between the OB and the vHPC–mPFC circuit on trials in which the rats subsequently made the successful working performance. Oscillatory OB activities were highly coherent with vHPC and mPFC in delta and gamma range associated with correct trials of the spatial working memory task. In addition, the delta phase had a more modulatory effect on the gamma power of the OB–vHPC–mPFC network during these correct trials. Moreover, synchrony between OB and vHPC–mPFC circuit in delta frequency enhanced when animals correctly performed the task. Here, we identified the contribution of OB in the vHPC–mPFC circuit in association with successful working memory performance.

Working memory is correlated with vHPC–mPFC circuit activity [3]. Lesioning studies indicated that the HPC–PFC interactions are essential for successful task performance [54]. Moreover, we previously reported that reduced correct responses within the working memory task correlate with disruption of the vHPC–mPFC circuit in pathologic conditions [55]. Consistent with previous studies, our results support the idea that enhancing coherence and correlation between vHPC and mPFC could be associated with optimized working memory performance, particularly leading to correct choices in a spatial working memory task. On the other hand, mPFC and vHPC anatomically and functionally are connected to OB [30, 31, 56].

OB oscillations are known as brain rhythms essential for synchronizing network activity during a cognitive task [31]. These rhythms are phase-locked to respiration and generated by triggering OSNs via air passage during nasal breathing, regardless of odor stimuli [32]. Moreover, it has been suggested that these respiratory-entrained rhythms generated by nasal respiration are global and visible during exploration, sleep REM phase, and air sampling through sniffing [57]. We previously demonstrated OB stimulation with nasal airflow that entrains oscillatory activity, particularly at delta frequencies in mPFC and vHPC [30]. Delta oscillations in OB have been reported to modulate brain oscillations during a cognitive performance, such as fear [29] and anxiety-like behavior [23]. Altogether, our findings suggest a functional connection between OB and vHPC–mPFC circuit at the delta band is associated with successful spatial working memory performance, indicating an optimized brain state during working memory.

According to human studies, gamma-band oscillations are tremendously involved in working memory [58, 59]. Moreover, in animals, a phase-locking of neural units in mPFC with vHPC gamma rhythm supports spatial encoding in working memory [8]. Analyzing LFPs and spiking activity of the PFC in monkeys also demonstrated that neural bursts in gamma oscillations (45–100 Hz) were associated with encoding and retrieval of sensory information during a working memory task [60]. Consistent with previous evidence, we observed that the coherence increment in the mPFC–vHPC circuit at gamma oscillations was associated with correct trials of spatial working memory tasks. We indicated that the coherent activity of OB with this circuit at the gamma band (50–80 Hz) was increased when animals correctly performed the task. Pharmacological inhibition or lesioning studies can help elucidate the significance of OB connection with brain circuits during working memory performance.

Growing evidence links the low–high frequency coupling with behavioral performance [6, 61, 62]. The present study demonstrated that delta–gamma coupling in vHPC–mPFC was significantly higher during correct trials than wrong working memory trials. We evaluated whether OB oscillations modulate the vHPC–mPFC circuit activity during the spatial working memory task. The results revealed that the OB delta phase modulates mPFC gamma power, and OB gamma power depends on the vHPC delta phase during correct trials. These observations confirm our hypothesis that OB can coordinate the vHPC–mPFC circuit during a cognitive task. The remaining question is how OB would drive these changes in vHPC–mPFC? Studies in awake rodents demonstrated that delta oscillations in the brain were phase-locked to respiration, known as respiration-entrained brain oscillation [56]. These oscillations are crucial for synchronizing information processing and network interaction during cognitive functions [63]. The respiration rhythm phase can modulate the gamma (30–80 Hz) oscillations power, and removing the OB abolishes respiration-locked delta oscillations and delta–gamma phase–amplitude coupling in the widespread brain regions [1].

Furthermore, respiration-entrained brain oscillations are suggested that functionally modulate delta, theta, and gamma activity in the mPFC [1, 42]. Moreover, HRR at the near-delta range (2–4 Hz) in the hippocampus was highly coherent with nasal respiration and rhythmic field potentials in the OB [36]. The HRR is coupled with gamma oscillations and diminishes when nasal airflow is eliminated, e.g., by tracheotomy [36]. However, we indicated that in the context of spatial working memory, OB activity might contribute to the vHPC–mPFC circuit activity modulation contributing to correct performance. To our knowledge, this is the first demonstration of the OB modulatory effects on the vHPC–mPFC circuit during spatial working memory performance. Given that OB activities potentially reflect changes in nasal breathing rhythm, we suggest future studies for recording nasal breathing simultaneously with working memory performance. This approach helps to examine how OB activity is affected by respiration rhythm and OSNs activities and whether nasal breathing is responsible for the OB modulatory role on the vHPC–mPFC circuit during working memory performance.

## Conclusions

We indicated that oscillatory activity of OB could coordinate vHPC–mPFC circuit activity during spatial working memory performance. Our results demonstrated that the interaction between delta and gamma oscillations of the OB–vHPC–mPFC network is associated with the successful performance of spatial working memory tasks. Our results revealed an interplay between these regions in an optimized brain state during a cognitive task. However, further studies are needed to uncover how OB influences the brain circuits during working memory performance.

### Supplementary Information

**Additional file 1: Figure S1.** Coherence in OB–vHPC–mPFC network. Lines display coherence from delta to gamma (0–120 Hz). The shaded area indicates standard errors, and gray areas show significant differences between correct and wrong trials. More noticeable changes are illustrated at < 4 Hz and 50–80 Hz. Data were analyzed by Wilcoxson-test, n = 6 per group. OB, olfactory bulb; mPFC, medial prefrontal cortex; vHPC, ventral hippocampus.

**Additional file 2: Figure S2.** Cross-frequency coupling between OB vHPC **(A)** Polar distribution. **(B)** Color map of gamma vHPC power (50–80 Hz) and delta phase cycle in OB. The green arrow denotes the mean resultant vector length. **(C)** Mean resultant vector length values show no significant differences as an indicator of the OB delta phase and vHPC gamma power coupling. Data were analyzed by the Wilcoxson test. OB, olfactory bulb; mPFC, medial prefrontal cortex; vHPC, ventral hippocampus.

## Data Availability

The data sets used and analyzed during the current study are available from the corresponding. author on reasonable request.

## References

[1] Wang G-W, Cai J-X (2006). Disconnection of the hippocampal–prefrontal cortical circuits impairs spatial working memory performance in rats. Behav Brain Res.

[2] Pickering C, Alsiö J, Morud J, Ericson M, Robbins TW, Söderpalm B (2015). Ethanol impairment of spontaneous alternation behaviour and associated changes in medial prefrontal glutamatergic gene expression precede putative markers of dependence. Pharmacol Biochem Behav.

[3] Tamura M, Spellman TJ, Rosen AM, Gogos JA, Gordon JA (2017). Hippocampal–prefrontal theta-gamma coupling during performance of a spatial working memory task. Nat Commun.

[4] Siapas AG, Lubenov EV, Wilson MA (2005). Prefrontal phase locking to hippocampal theta oscillations. Neuron.

[5] Jin J, Maren S (2015). Prefrontal–hippocampal interactions in memory and emotion. Front Syst Neurosci.

[6] Tort ABL, Komorowski RW, Manns JR, Kopell NJ, Eichenbaum H (2009). Theta—gamma coupling increases during the learning of item—context associations. Proc Natl Acad Sci.

[7] Xia M, Liu T, Bai W, Zheng X, Tian X (2019). Information transmission in HPC–PFC network for spatial working memory in rat. Behav Brain Res.

[8] Spellman T, Rigotti M, Ahmari SE, Fusi S, Gogos JA, Gordon JA (2015). Hippocampal–prefrontal input supports spatial encoding in working memory. Nature.

[9] Kaplan R, Bush D, Bisby JA, Horner AJ, Meyer SS, Burgess N (2017). Medial prefrontal–medial temporal theta phase coupling in dynamic spatial imagery. J Cogn Neurosci.

[10] Krieger-Redwood K, Jefferies E, Karapanagiotidis T, Seymour R, Nunes A, Ang JWA, Majernikova V, Mollo G, Smallwood J (2016). Down but not out in posterior cingulate cortex: deactivation yet functional coupling with prefrontal cortex during demanding semantic cognition. Neuroimage.

[11] Viejo G, Peyrache A (2020). Precise coupling of the thalamic head-direction system to hippocampal ripples. Nat Commun.

[12] Pina JE, Bodner M, Ermentrout B (2018). Oscillations in working memory and neural binding: a mechanism for multiple memories and their interactions. PLoS Comput Biol.

[13] Klimesch W (1999). EEG alpha and theta oscillations reflect cognitive and memory performance: a review and analysis. Brain Res Brain Res Rev.

[14] Herrmann CS, Strüber D, Helfrich RF, Engel AK (2016). EEG oscillations: from correlation to causality. Int J Psychophysiol.

[15] Wang XJ (2010). Neurophysiological and computational principles of cortical rhythms in cognition. Physiol Rev.

[16] Hasselmo ME, Bodelón C, Wyble BP (2002). A proposed function for hippocampal theta rhythm: separate phases of encoding and retrieval enhance reversal of prior learning. Neural Comput.

[17] Colgin LL (2011). Oscillations and hippocampal–prefrontal synchrony. Curr Opin Neurobiol.

[18] Hyman JM, Hasselmo ME, Seamans JK (2011). What is the functional relevance of prefrontal cortex entrainment to hippocampal theta rhythms?. Front Neurosci.

[19] Liu T, Bai W, Xia M, Tian X (2018). Directional hippocampal-prefrontal interactions during working memory. Behav Brain Res.

[20] Salimi M, Tabasi F, Nazari M, Ghazvineh S, Salimi A, Jamaati H, Raoufy MR (2021). The olfactory bulb modulates entorhinal cortex oscillations during spatial working memory. J Physiol Sci.

[21] Merrick C, Godwin C, Geisler M, Morsella E (2014). The olfactory system as the gateway to the neural correlates of consciousness. Front Psychol.

[22] Yaldizli Ö, Penner IK, Yonekawa T, Naegelin Y, Kuhle J, Pardini M, Chard DT, Stippich C, Kira JI, Bendfeldt K, Amann M, Radue EW, Kappos L, Sprenger T (2016). The association between olfactory bulb volume, cognitive dysfunction, physical disability and depression in multiple sclerosis. Eur J Neurol.

[23] Salimi M, Ghazvineh S, Zare M, Parsazadegan T, Dehdar K, Nazari M, Mirnajafi-Zadeh J, Jamaati H, Raoufy MR (2019). Distraction of olfactory bulb-medial prefrontal cortex circuit may induce anxiety-like behavior in allergic rhinitis. PLoS ONE.

[24] Arshamian A, Iravani B, Majid A, Lundström JN (2018). Respiration modulates olfactory memory consolidation in humans. J Neurosci.

[25] Zelano C, Jiang H, Zhou G, Arora N, Schuele S, Rosenow J, Gottfried JA (2016). Nasal respiration entrains human limbic oscillations and modulates cognitive function. J Neurosci.

[26] Heck DH, Kozma R, Kay LM (2019). The rhythm of memory: how breathing shapes memory function. J Neurophysiol.

[27] Vanderwolf C (1992). Hippocampal activity, olfaction, and sniffing: an olfactory input to the dentate gyrus. Brain Res.

[28] Van Groen T, Wyss JM (1990). Extrinsic projections from area CA1 of the rat hippocampus: olfactory, cortical, subcortical, and bilateral hippocampal formation projections. J Comp Neurol.

[29] Moberly AH, Schreck M, Bhattarai JP, Zweifel LS, Luo W, Ma M (2018). Olfactory inputs modulate respiration-related rhythmic activity in the prefrontal cortex and freezing behavior. Nat Commun.

[30] Ghazvineh S, Salimi M, Nazari M, Garousi M, Tabasi F, Dehdar K, Salimi A, Jamaati H, Mirnajafi-Zadeh J, Arabzadeh E, Raoufy MR (2021). Rhythmic air-puff into nasal cavity modulates activity across multiple brain areas: A non-invasive brain stimulation method to reduce ventilator-induced memory impairment. Respir Physiol Neurobiol.

[31] Tort ABL, Brankačk J, Draguhn A (2018). Respiration-entrained brain rhythms are global but often overlooked. Trends Neurosci.

[32] Grosmaitre X, Santarelli LC, Tan J, Luo M, Ma M (2007). Dual functions of mammalian olfactory sensory neurons as odor detectors and mechanical sensors. Nat Neurosci.

[33] Salimi M, Tabasi F, Ghazvineh S, Jamaati H, Salimi A, Raoufy MR (2022). Stimulating neural pathways to reduce mechanical ventilation-associated neurocognitive dysfunction. Am J Respir Crit Care Med.

[34] Salimi M, Javadi A-H, Nazari M, Bamdad S, Tabasi F, Parsazadegan T, Ayene F, Karimian M, Gholami-Mahtaj L, Shadnia S, Jamaati H, Salimi A, Raoufy MR (2022). Nasal Air Puff Promotes Default Mode Network Activity in Mechanically Ventilated Comatose Patients: a Noninvasive Brain Stimulation Approach. Neuromodulation.

[35] Heck DH, McAfee SS, Liu Y, Babajani-Feremi A, Rezaie R, Freeman WJ, Wheless JW, Papanicolaou AC, Ruszinkó M, Sokolov Y, Kozma R (2017). Breathing as a fundamental rhythm of brain function. Front Neural Circuits.

[36] Yanovsky Y, Ciatipis M, Draguhn A, Tort AB, Brankačk J (2014). Slow oscillations in the mouse hippocampus entrained by nasal respiration. J Neurosci.

[37] Lockmann AL, Laplagne DA, Leão RN, Tort AB (2016). A respiration-coupled rhythm in the rat hippocampus independent of theta and slow oscillations. J Neurosci.

[38] Chi VN, Müller C, Wolfenstetter T, Yanovsky Y, Draguhn A, Tort AB, Brankačk J (2016). Hippocampal respiration-driven rhythm distinct from theta oscillations in awake mice. J Neurosci.

[39] Gourévitch B, Kay LM, Martin C (2010). Directional coupling from the olfactory bulb to the hippocampus during a go/no-go odor discrimination task. J Neurophysiol.

[40] Fujisawa S, Buzsáki G (2011). A _4_ Hz oscillation adaptively synchronizes prefrontal, VTA, and hippocampal activities. Neuron.

[41] Ito J, Roy S, Liu Y, Cao Y, Fletcher M, Lu L, Boughter J, Grün S, Heck D (2014). Whisker barrel cortex delta oscillations and gamma power in the awake mouse are linked to respiration. Nat Commun.

[42] Biskamp J, Bartos M, Sauer J-F (2017). Organization of prefrontal network activity by respiration-related oscillations. Sci Rep.

[43] Hozumi S, Nakagawasai O, Tan-No K, Niijima F, Yamadera F, Murata A, Arai Y, Yasuhara H, Tadano T (2003). Characteristics of changes in cholinergic function and impairment of learning and memory-related behavior induced by olfactory bulbectomy. Behav Brain Res.

[44] van Rijzingen IM, Gispen WH, Spruijt BM (1995). Olfactory bulbectomy temporarily impairs Morris maze performance: an ACTH (4–9) analog accellerates return of function. Physiol Behav.

[45] Yamamoto T, Jin J, Watanabe S (1997). Characteristics of memory dysfunction in olfactory bulbectomized rats and the effects of cholinergic drugs. Behav Brain Res.

[46] Adrian E (1951). The role of air movement in olfactory stimulation. J Physiol.

[47] Paxinos G, Watson C (2006). The rat brain in stereotaxic coordinates: hard cover.

[48] Delorme A, Makeig S (2004). EEGLAB: an open source toolbox for analysis of single-trial EEG dynamics including independent component analysis. J Neurosci Methods.

[49] Thierry AM, Gioanni Y, Dégénétais E, Glowinski J (2000). Hippocampo-prefrontal cortex pathway: anatomical and electrophysiological characteristics. Hippocampus.

[50] Friese U, Köster M, Hassler U, Martens U, Trujillo-Barreto N, Gruber T (2013). Successful memory encoding is associated with increased cross-frequency coupling between frontal theta and posterior gamma oscillations in human scalp-recorded EEG. Neuroimage.

[51] Rochart R, Liu Q, Fonteh AN, Harrington MG, Arakaki X (2020). Compromised behavior and gamma power during working memory in cognitively healthy individuals with abnormal CSF amyloid/tau. Front Aging Neurosci.

[52] Hallock HL, Wang A, Griffin AL (2016). Ventral midline thalamus is critical for hippocampal–prefrontal synchrony and spatial working memory. J Neurosci.

[53] Dehdar K, Mahdidoust S, Salimi M, Gholami-Mahtaj L, Nazari M, Mohammadi S, Dehghan S, Jamaati H, Khosrowabadi R, Nasiraei-Moghaddam A (2019). Allergen-induced anxiety-like behavior is associated with disruption of medial prefrontal cortex-amygdala circuit. Sci Rep.

[54] Izaki Y, Takita M, Akema T (2008). Specific role of the posterior dorsal hippocampus–prefrontal cortex in short-term working memory. Eur J Neurosci.

[55] Salimi M, Ghazvineh S, Nazari M, Dehdar K, Garousi M, Zare M, Tabasi F, Jamaati H, Salimi A, Barkley V (2021). Allergic rhinitis impairs working memory in association with drop of hippocampal–prefrontal coupling. Brain Res.

[56] Lockmann AL, Tort AB (2018). Nasal respiration entrains delta-frequency oscillations in the prefrontal cortex and hippocampus of rodents. Brain Struct Funct.

[57] Zhong W, Ciatipis M, Wolfenstetter T, Jessberger J, Müller C, Ponsel S, Yanovsky Y, Brankačk J, Tort ABL, Draguhn A (2017). Selective entrainment of gamma subbands by different slow network oscillations. Proc Natl Acad Sci USA.

[58] Howard MW, Rizzuto DS, Caplan JB, Madsen JR, Lisman J, Aschenbrenner-Scheibe R, Schulze-Bonhage A, Kahana MJ (2003). Gamma oscillations correlate with working memory load in humans. Cereb Cortex.

[59] Basar-Eroglu C, Brand A, Hildebrandt H, Kedzior KK, Mathes B, Schmiedt C (2007). Working memory related gamma oscillations in schizophrenia patients. Int J Psychophysiol.

[60] Lundqvist M, Rose J, Herman P, Brincat SL, Buschman TJ, Miller EK (2016). Gamma and beta bursts underlie working memory. Neuron.

[61] Stujenske JM, Likhtik E, Topiwala MA, Gordon JA (2014). Fear and safety engage competing patterns of theta-gamma coupling in the basolateral amygdala. Neuron.

[62] Brooks H, Goodman MS, Bowie CR, Zomorrodi R, Blumberger DM, Butters MA, Daskalakis ZJ, Fischer CE, Flint A, Herrmann N (2020). Theta—gamma coupling and ordering information: a stable brain–behavior relationship across cognitive tasks and clinical conditions. Neuropsychopharmacology.

[63] Vos T, Abajobir AA, Abate KH, Abbafati C, Abbas KM, Abd-Allah F, Abdulkader RS, Abdulle AM, Abebo TA, Abera SF (2017). Global, regional, and national incidence, prevalence, and years lived with disability for 328 diseases and injuries for 195 countries, 1990–2016: a systematic analysis for the Global Burden of Disease Study 2016. Lancet.

