# Supplementary figures and images for: The olfactory bulb coordinates the ventral hippocampus–medial prefrontal cortex circuit during spatial working memory performance
Source: J Physiol Sci. 2022 Apr 25;72:9. doi: 10.1186/s12576-022-00833-5 (PMC10717655; doi:10.1186/s12576-022-00833-5)

# OB phase-vHPC power

A

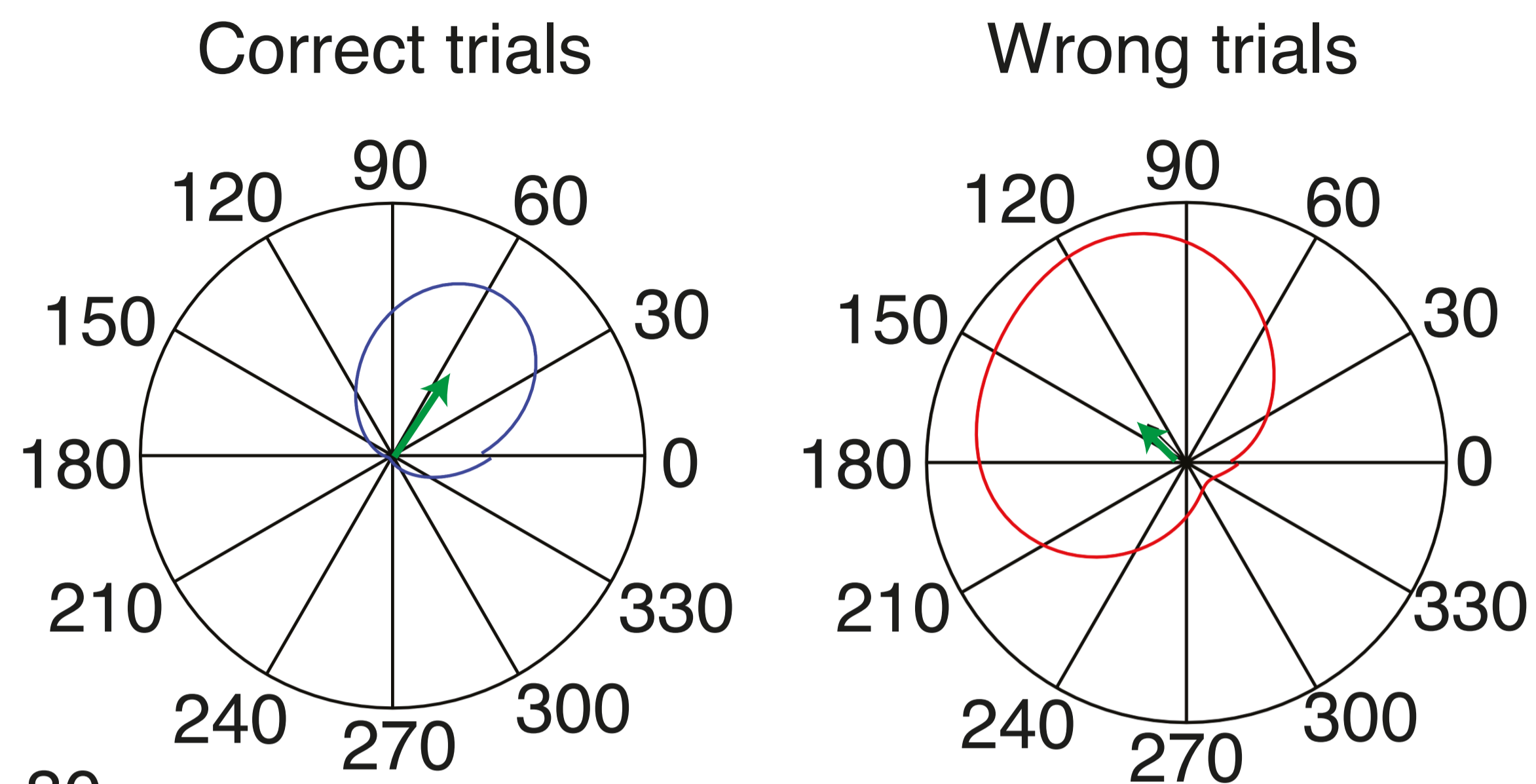

B

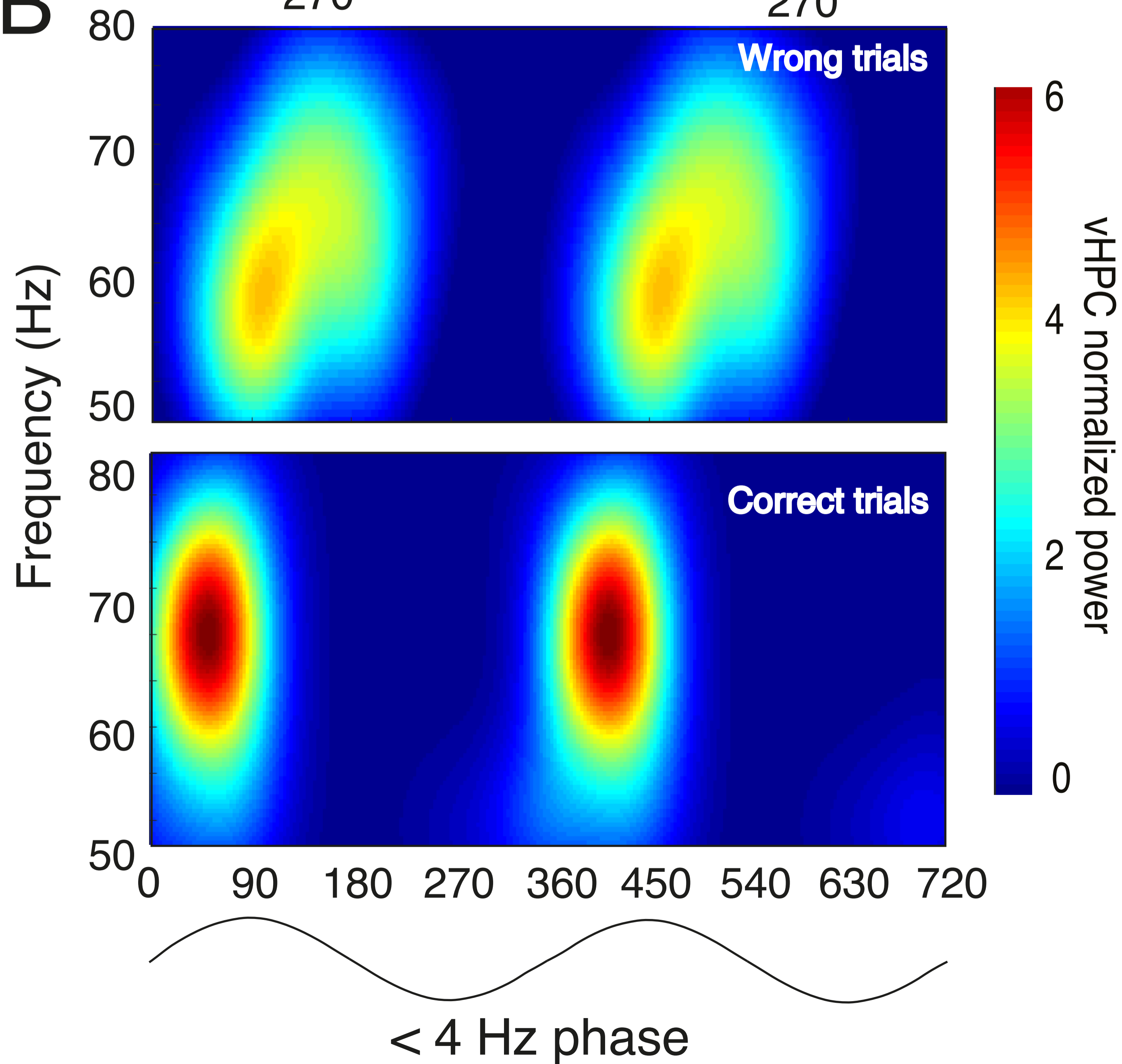

C

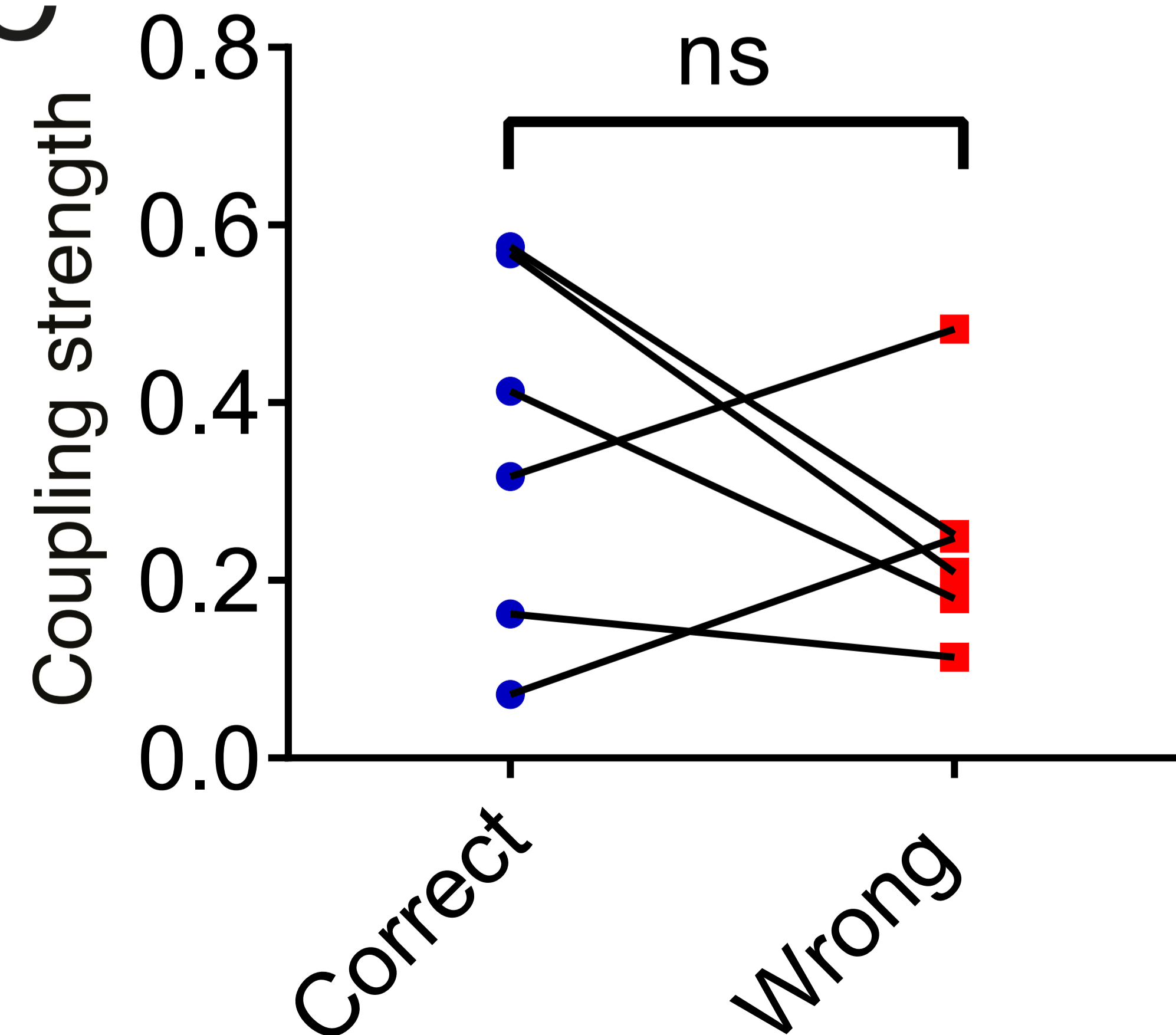

Supplement: Supplementary file 2 — Additional file 2: Figure S2. Cross-frequency coupling between OB vHPC (A) Polar distribution. (B) Color map of gamma vHPC power (50–80 Hz) and delta phase cycle in OB. The green arrow denotes the mean resultant vector length. (C) Mean resultant vector length values show no significant differences as an indicator of the OB delta phase and vHPC gamma power coupling. Data were analyzed by the Wilcoxson test. OB, olfactory bulb; mPFC, medial prefrontal cortex; vHPC, ventral hippocampus. [file 12576_2022_833_MOESM2_ESM.pdf]
