# Supplementary material for: The olfactory bulb coordinates the ventral hippocampus–medial prefrontal cortex circuit during spatial working memory performance
Source: J Physiol Sci. 2022 Apr 25;72:9. doi: 10.1186/s12576-022-00833-5 (PMC10717655; doi:10.1186/s12576-022-00833-5)
Supplement: Supplementary file 1 — Additional file 1: Figure S1. Coherence in OB–vHPC–mPFC network. Lines display coherence from delta to gamma (0–120 Hz). The shaded area indicates standard errors, and gray areas show significant differences between correct and wrong trials. More noticeable changes are illustrated at < 4 Hz and 50–80 Hz. Data were analyzed by Wilcoxson-test, n = 6 per group. OB, olfactory bulb; mPFC, medial prefrontal cortex; vHPC, ventral hippocampus. [file 12576_2022_833_MOESM1_ESM.pdf]

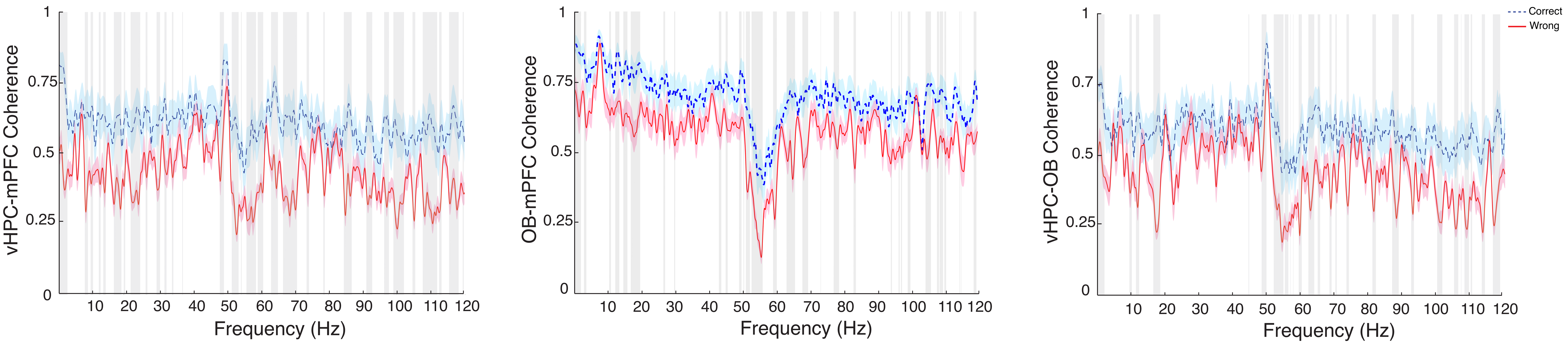
vHPC-mPFC Coherence
OB-mPFC Coherence
vHPC-OB Coherence
Frequency (Hz)
Frequency (Hz)
Frequency (Hz)
Correct
Wrong
0
0.25
0.5
0.75
1
0.7
10 20 30 40 50 60 70 80 90 100 110 120
